# CC-Chemokine Ligand 2 (CCL2) Suppresses High Density Lipoprotein (HDL) Internalization and Cholesterol Efflux via CC-Chemokine Receptor 2 (CCR2) Induction and p42/44 Mitogen-activated Protein Kinase (MAPK) Activation in Human Endothelial Cells *

**DOI:** 10.1074/jbc.M116.714279

**Published:** 2016-07-25

**Authors:** Run-Lu Sun, Can-Xia Huang, Jin-Lan Bao, Jie-Yu Jiang, Bo Zhang, Shu-Xian Zhou, Wei-Bin Cai, Hong Wang, Jing-Feng Wang, Yu-Ling Zhang

**Affiliations:** From the ‡ Cardiovascular Medicine Department,; § Intensive Care Unit, and; ¶ Comprehensive Department, Sun Yat-sen Memorial Hospital, Sun Yat-sen Memorial Hospital, Sun Yat-sen University, Guangzhou 510120, China,; the ‖ Graceland Medical Center, the Sixth Affiliated Hospital, Sun Yat-sen University, Guangzhou 510655, China,; the ** Guangdong Province Key Laboratory of Arrhythmia and Electrophysiology, Guangzhou 51020, China,; the ‡‡ Department of Biochemistry, Zhongshan Medical School, Sun Yat-sen University, Guangzhou 510080, China, and; the §§ Centers for Metabolic and Cardiovascular Research, Departments of Pharmacology, Temple University, Philadelphia, Pennsylvania 19140

**Keywords:** atherosclerosis, cholesterol metabolism, endothelial cell, high-density lipoprotein (HDL), mitogen-activated protein kinase (MAPK), CC-chemokine ligand 2 (CCL2)

## Abstract

High density lipoprotein (HDL) has been proposed to be internalized and to promote reverse cholesterol transport in endothelial cells (ECs). However, the mechanism underlying these processes has not been studied. In this study, we aim to characterize HDL internalization and cholesterol efflux in ECs and regulatory mechanisms. We found mature HDL particles were reduced in patients with coronary artery disease (CAD), which was associated with an increase in CC-chemokine ligand 2 (CCL2). In cultured primary human coronary artery endothelial cells and human umbilical vein endothelial cells, we determined that CCL2 suppressed the binding (4 °C) and association (37 °C) of HDL to/with ECs and HDL cellular internalization. Furthermore, CCL2 inhibited [^3^H]cholesterol efflux to HDL/apoA1 in ECs. We further found that CCL2 induced CC-chemokine receptor 2 (CCR2) expression and siRNA-*CCR2* reversed CCL2 suppression on HDL binding, association, internalization, and on cholesterol efflux in ECs. Moreover, CCL2 induced p42/44 mitogen-activated protein kinase (MAPK) phosphorylation via CCR2, and p42/44 MAPK inhibition reversed the suppression of CCL2 on HDL metabolism in ECs. Our study suggests that CCL2 was elevated in CAD patients. CCL2 suppressed HDL internalization and cholesterol efflux via CCR2 induction and p42/44 MAPK activation in ECs. CCL2 induction may contribute to impair HDL function and form atherosclerosis in CAD.

## Introduction

Plasma HDL cholesterol (HDL-C) ^3^ concentrations exhibit an inverse correlation with the incidence of coronary artery disease (1). However, to date, pharmacologically raising HDL-C levels has not been demonstrated to prevent atherosclerosis (2). Therefore, the mechanisms by which HDL may impact cardiovascular disease remain complex and not fully understood (3). The crucial cardioprotective function of HDL in reverse cholesterol transport (RCT) is removing cholesterol from macrophages in the arterial intima and delivering it to the liver for excretion into the bile (4). Interestingly, an early step of RCT is the transfer of cellular cholesterol from macrophages to HDL, which occurs in the subendothelial space of arteries rather than in the plasma during the progression of atherosclerosis (5). Therefore, HDL must cross the endothelium to move into close proximity to cholesterol donor cells and to facilitate cholesterol efflux. This process includes HDL binding, cell association, internalization, and transport by vascular endothelial cells. This transendothelial macromolecule translocation of HDL is similar to that of apoA-1 and involves SR-BI and ABCG1 (6). Well beyond the direct modulation of HDL on endothelial cell apoptosis, proliferation and migration, and anti-inflammatory activity, cholesterol efflux in endothelial cells plays a vital vascular protective role by minimizing the amount of cholesterol entering the vessel intima (7).

CCL2 is a member of the CC chemokine family that is highly involved in the initiation and progression of atherosclerosis (8–10). A few large cohort studies (11, 12) have shown that CCL2 might mediate the pro-atherogenic effects of dyslipidemia. CC-chemokine receptor 2 (CCR2), CCR3, and CCR5, which are high affinity receptors for CCL2 (13), are expressed on human endothelial cells (14–16). A pathogenic role of CCR2 in atherosclerosis has been clearly established through evidence of a sustained reduction in atherosclerotic lesions in ccr2-deficient mice (17). Furthermore, ccr5 deficiency imposes restrictions on atherosclerotic plaque formation in atherosclerosis-prone mice by reducing the systemic immuno-inflammatory response and contributing to large changes in lesion composition during the initial stages of plaque development (18, 19).

The capacity of RCT was shown to be impaired during low degree inflammation in the progression of atherosclerosis, but little is known regarding how CCL2 influences the early step of RCT together with HDL internalization and cholesterol efflux to HDL in ECs. HCAECs form a special endothelial lining that functions as a semipermeable barrier of coronary arteries. Disorders of cholesterol efflux in ECs may result in unrestricted accumulation of cellular cholesterol and prevent the foam cell phenotype change observed in macrophages in atherosclerotic plaques. In our previous study (20), CCL2 impaired RCT activity by PI3K/Akt-mediated post-translational regulation of ABCA1, ABCG1, and SR-BI cell surface expression in HepG2 cells and primary liver cells. In this study, we found that plasma CCL2 levels were elevated and negatively correlated with mature HDL-C levels in patients with CAD. Next, we investigated the impact of pro-atherogenic effects of CCL2, together with its receptors, on the initial steps of RCT in ECs, *i.e.* crossing the endothelium, HDL binding, association, internalization, and cholesterol efflux in cultured HCAECs and HUVECs, as well as the regulatory mechanisms.

## Results

### 

#### 

##### Plasma CCL2 Levels and Lipid Profiles in Patients with CAD

The demographic and clinical characteristics of CAD patients and healthy controls are shown in Table 1A. Regarding the HDL-C and CCL2 levels in the two groups, the patients with CAD had higher CCL2 levels (*p* = 0.001) and lower HDL-C levels (*p* < 0.001) compared with the healthy controls. Moreover, HDL2 and apoA1 levels were decreased, and non-HDL-C levels were increased in CAD patients compared with the healthy controls (Table 1B). Furthermore, we found CCL2 was negatively correlated with HDL2 but not correlated with HDL-C, apoA1, TC, TG, and LDL-C in CAD patients by regression analysis (Table 1, C–H). After adjusting sex, age, BMI, smoking, drinking, history of CHD, hypertension, and diabetes or HDL-C, apoA1, TC, TG, and LDL-C, CCL2 was still negatively correlated with HDL2 (Table 1I).

**TABLE 1:**
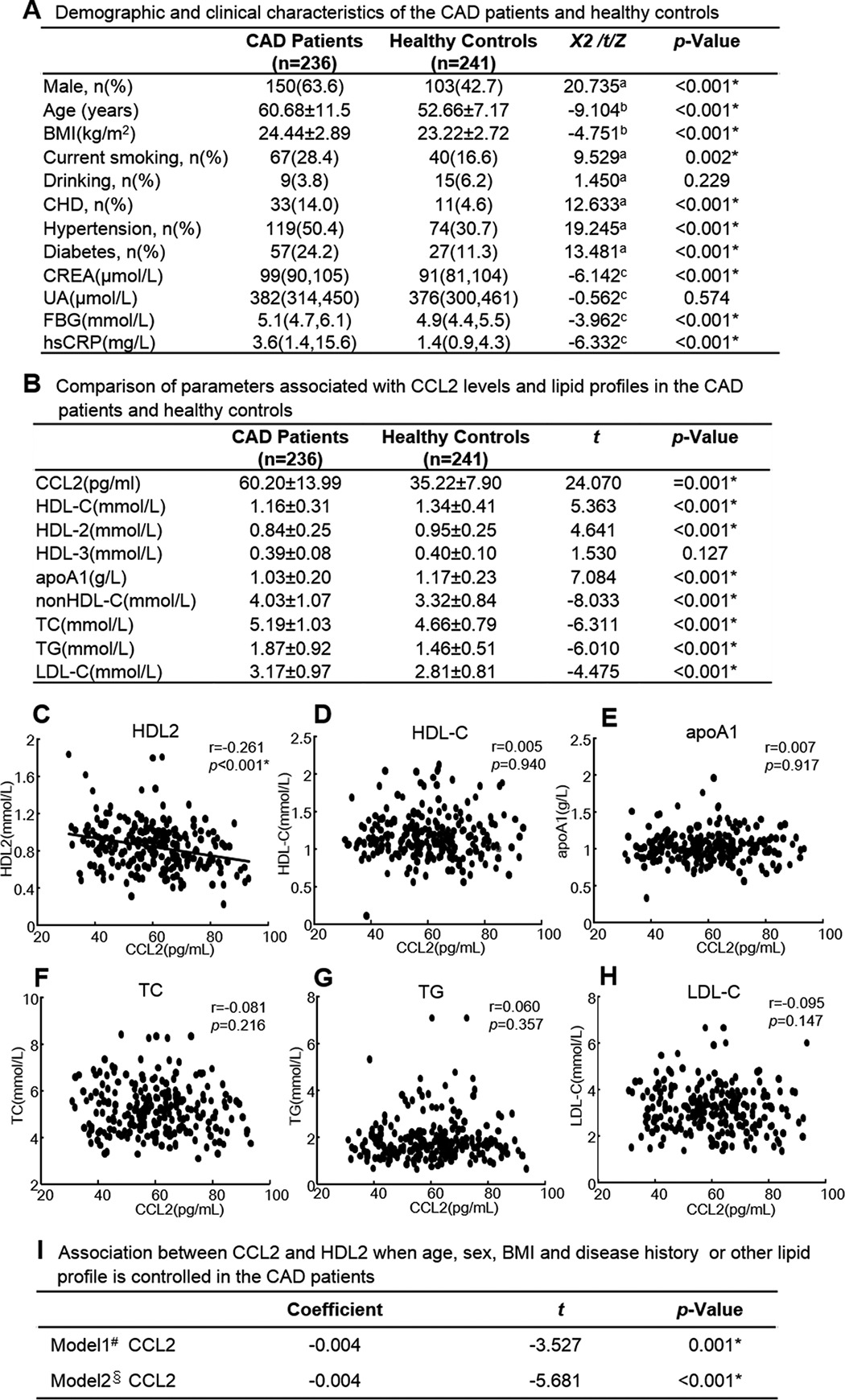
**Plasma CCL2 level was negatively correlated with HDL2 in CAD patients** Blood from CAD patients was drawn after an overnight fast and collected in EDTA-coated tubes. An Olympus AU-640 (Germany) was used for HDL-C, apoA1, TC, TG, LDL-C analysis, and ELISA (BMS281TEN, Austria) was used for measuring CCL2. The levels of HDL particles were measured as described under “Experimental Procedures.” The simple correlation and multiple regression after adjustment of potential confounders was performed to analysis the association between CCL2 and lipid profile by SPSS 14.0 software. Each data point represents one patient. A, demographic and clinical characteristics of the CAD patients and healthy controls. B, comparison of parameters associated with CCL2 levels and lipid profiles in the CAD patients and healthy controls. C, correlation of CCL2 with HDL2 in CAD patients. D, correlation of CCL2 with HDL-C in CAD patients. E, correlation of CCL2 with apoA1 in CAD patients. F, correlation of CCL2 with TC in CAD patients. G, correlation of CCL2 with TG in CAD patients. H, correlation of CCL2 with LDL-C in CAD patients. I, association between CCL2 and HDL2 when age, sex, BMI and disease history or other lipid profile are controlled in the CAD patients. Continuous variables were expressed as the means ± S.D. (for normally distributed variables) or medians with interquartile ranges (for non-normally distributed variables). Categorical variables were expressed as case numbers (percentages). BMI, body mass index; CHD, coronary heart disease; CREA, Creatinine; UA, uric acid; FBG, fasting blood glucose; hsCRP, high sensitivity C-reactive protein; CCL2, CC chemokine ligand; HDL-C, high density lipoprotein-cholesterol; apoA1, apolipoprotein A1; TC, total cholesterol; TG, triglyceride; LDL-C, low density lipoprotein cholesterol. *^a^* represents the χ^2^ statistic; *^b^* represents the *t* statistic; *^c^* represents Z statistic. # adjusted for sex, age, BMI, history of smoking, drinking, CHD, hypertension and diabetes. §, adjusted for HDL-C, apoA1, TC, TG, LDL-C. *, *P*<0.05 as significance.

##### CCL2 Suppressed HDL Binding, Association, and Internalization in HCAECs and HUVECs

**FIGURE 1.**
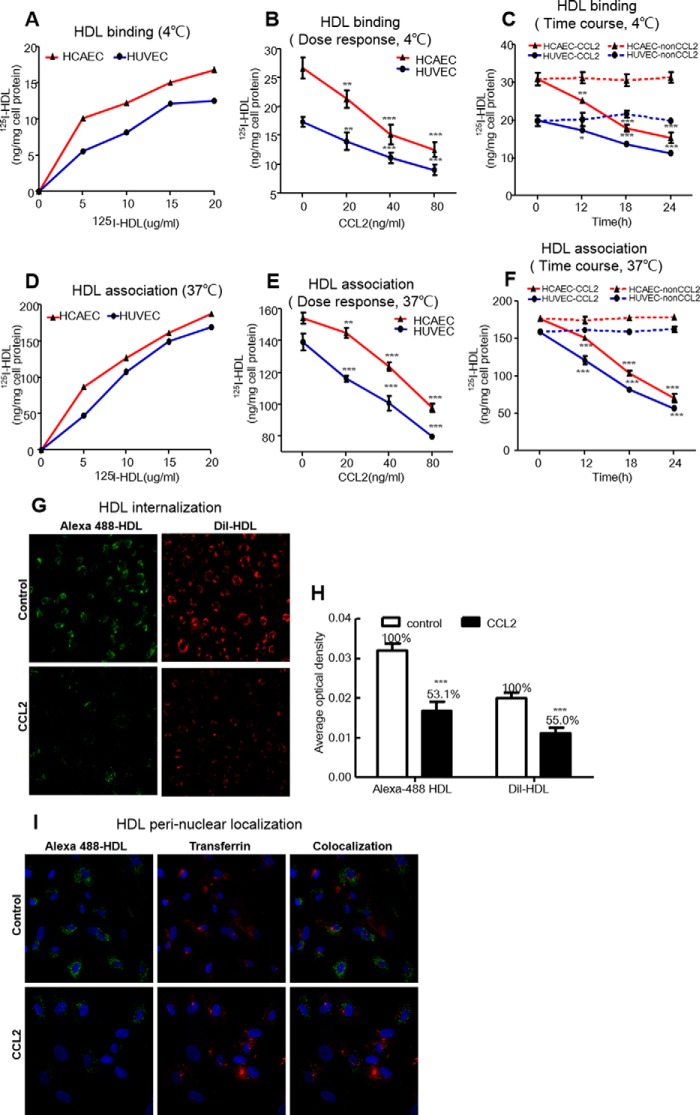
**CCL2 suppressed the binding (4 °C) and association (37 °C) of HDL to/with ECs, and HDL cellular internalization.** *A,* binding of ^125^I-HDL to ECs at 4 °C. HCAECs and HUVECs were incubated with the indicated concentration of ^125^I-HDL (0, 5, 10, 15, and 20 μg/ml) for 1 h, in the absence (total binding) or in the presence of a 40-fold excess of unlabeled HDL (nonspecific binding) at 4 °C. Then the cells were solubilized in 0.1 n NaOH for 60 min, and the protein concentration and radioactivity were measured in the lysate. Radioactivity was measured by using a counter from PerkinElmer Life Sciences, and the measurements were normalized to the protein content determined by the Bradford protein assay (Bio-Rad). Specific binding was calculated by subtracting the values of the unspecific binding from those of the total binding. *B,* dose response of CCL2 on HDL binding. Cells were incubated with 10 μg/ml ^125^I-HDL at 4 °C for 1 h after treating with increasing doses of CCL2 (0, 20, 40, and 80 ng/ml) for 18 h. Other procedures were the same as *A. C,* time course of CCL2 on HDL binding. Cells were incubated with 10 μg/ml ^125^I-HDL at 4 °C for 1 h after treating with CCL2 at 40 ng/ml for the indicated times (0, 12, 18, and 24 h). Other procedures were the same as *A. D,* association of ^125^I-HDL with ECs at 37 °C. HCAECs and HUVECs were incubated with the indicated concentrations of ^125^I-HDL (0, 5, 10, 15, and 20 μg/ml) for 1 h in the absence or in the presence of a 40-fold excess of unlabeled HDL at 37 °C. Other procedures were the same as *A. E,* dose response of CCL2 on HDL association. Cells were incubated with 10 μg/ml ^125^I-HDL at 37 °C for 1 h after treating with increasing doses of CCL2 (0, 20, 40, and 80 ng/ml) for 18 h. Other procedures were the same as *A. F,* time course of CCL2 on HDL association. Cells were incubated with 10 μg/ml ^125^I-HDL at 37 °C for 1 h after treating with CCL2 at 40 ng/ml for the indicated times (0, 12, 18, and 24 h). Other procedures were the same as *A. G,* effect of CCL2 on HDL internalization. Live ECs were incubated with 10 μg/ml Alexa 488-HDL (*green*)-labeled protein and DIL-HDL(*red*)-labeled phospholipid for 1 h at 37 °C after treating with 40 ng/ml CCL2 for18 h. The cells were fixed and imaged using a confocal microscope (LSM780) (×20). *H,* quantitative analysis of HDL internalization induced by CCL2. *I,* HDL perinuclear localization. Cells were incubated with Alexa 488-HDL (*green*) together with Alexa 594-transferrin (*red*) after treating with 40 ng/ml CCL2 for 18 h, and partial co-localization (*yellow*) was assessed. Cells were fixed and imaged using a confocal microscope (LSM780) (×20). The results are represented as the mean ± S.D. of at least three individual experiments performed in triplicate. *, *p* < 0.05; **, *p* < 0.01; ***, *p* < 0.001 compared with the untreated cells.

We next determined the effect of CCL2 on HDL uptake by ECs. This effect was characterized by determining ^125^I-HDL binding at 4 °C and cell association at 37 °C in live HCAECs and HUVECs. The specific binding of HDL at 4 °C and association at 37 °C to/with ECs were increased with the increasing doses of ^125^I-HDL (Fig. 1, *A* and *D*). ECs were treated with increasing doses of CCL2 (0, 20, 40, and 80 ng/ml) for 18 h or with increasing times (0, 12, 18, and 24 h) at a fixed dose of 40 ng/ml CCL2. CCL2 decreased ^125^I-HDL binding in HCAECs and HUVECs from 20.2 to 19.5% at 20 ng/ml up to 52.8 and 47.7% at 80 ng/ml (Fig. 1*B*) and from 19.2 to 13.9% at 12 h up to 51.0 and 43.2% at 24 h (Fig. 1*C*), respectively. Similarly, CCL2 also decreased ^125^I-HDL cell association in HCAECs and HUVECs from 6.1 to 16.6% at 20 ng/ml up to the maximum values of 36.6 and 42.7% at 80 ng/ml (Fig. 1*E*) and from 13.2 to 25.1% at 12 h up to the maximum values of 60.6 and 65.0% at 24 h (Fig. 1*F*), respectively. Because of the significance of the observed results, all subsequent experiments were performed with cells incubated with 40 ng/ml CCL2 for 18 h. We found TNF-α (10 ng/ml for 24 h) incubated with HCAECs could produce the maximum concentration of endogenous CCL2 (16,988 ± 276 pg/ml) but could not significantly inhibit the ^125^I-HDL binding and association to/with ECs (data not shown).

To investigate the effect of CCL2 on the internalization of HDL phospholipid and protein by HCAECs, we used DIL-HDL to follow the fate of HDL phospholipid and Alexa 488-HDL to visualize the HDL protein. We observed that the internalization of the HDL protein in HCAECs was accompanied by the uptake of HDL phospholipid. The internalization of DIL-HDL phospholipid and Alexa 488-HDL protein was decreased by 46.9 and 45.0%, respectively, and induced by 40 ng/ml CCL2 for 18 h (Fig. 1, *G* and *H*).

Furthermore, we used Alexa 594-transferrin incubated together with Alexa 488-HDL for tracing the internalized location of HDL protein and for observing the effect of CCL2. The vesicles containing green fluorescent HDL partially co-localized with red Alexa 594-transferrin in the perinuclear area, confirming that HDL was internalized by ECs. Using this method, we also observed that the internalization of HDL protein by HCAECs was significantly suppressed by CCL2 compared with untreated cells (Fig. 1*I*).

##### CCL2 Inhibited the [^3^H]Cholesterol Efflux to HDL/ApoA1 in HCAECs and HUVECs

Because the impaired capacity of cholesterol efflux to HDL/apoA1 from ECs may result in an accumulation of cellular cholesterol and lead to the forming of atherosclerotic plaque, we next explored whether CCL2 could inhibit the [^3^H]cholesterol efflux to HDL/apoA1 in both HCAECs and HUVECs.

**FIGURE 2.**
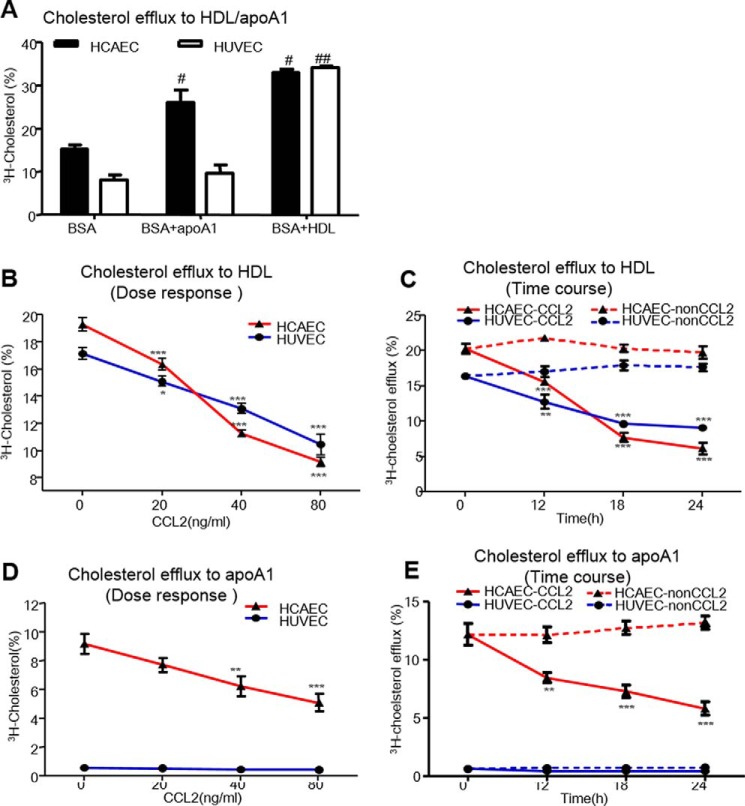
**CCL2 inhibited [^3^H]cholesterol efflux to HDL/apoA1 in ECs.** *A,* basal cholesterol efflux to HDL/apoA1 in ECs. HCAECs and HUVECs were loaded with [^3^H]cholesterol (1 Ci/ml) for 24 h. Efflux was initiated by BSA alone and BSA plus 20 μg/ml apoA1 or 50 μg/ml HDL for 2 h. The radioactivity of the medium and cells was measured with a liquid scintillation counter. The cholesterol efflux was expressed as the percentage of counts in the medium relative to the total counts for the medium and cells together. *B,* dose response of CCL2 on cholesterol efflux to HDL. Cells were initiated by 50 μg/ml human HDL 2 h after treating with increasing doses (0, 20, 40, and 80 ng/ml) of CCL2. The final cholesterol efflux was calculated as the percentage of total [^3^H]cholesterol released into the medium after subtraction of the values obtained in the absence of HDL. Other procedures are the same as *A. C,* time course of CCL2 on cholesterol efflux to HDL. Cells were incubated with 50 μg/ml human HDL 2 h after treating with 40 ng/ml CCL2 for indicate times (0, 12, 18, and 24 h). Others were the same as *A. D,* dose response of CCL2 on cholesterol efflux to apoA1. Cells were initiated by 20 μg/ml human apoA1 2 h after CCL2 treated with increasing doses. Other procedures were the same as *A. E,* time course of CCL2 on cholesterol efflux to apoA1. Cells were incubated by 20 μg/ml human apoA1 2 h after CCL2 treatment for different times. Other procedures were the same as *A*. The results were expressed as mean ± S.D. (*n* = 3). The points represent the averages of three values. #, *p* < 0.001 compared with the BSA alone in HCAEC; *##, p* < 0.001 compared with the BSA alone in HUVEC. *, *p* < 0.05; **, *p* < 0.01; ***, *p* < 0.001 compared with the untreated cells.

First, we observed that HCAECs had a high basal rate of [^3^H]cholesterol efflux to BSA compared with HUVECs, and 50 μg/ml HDL increased the [^3^H]cholesterol efflux from HCAECs and HUVECs by 54.1 and 76.5%, respectively. Nevertheless, 20 μg/ml apoA1 only increased the [^3^H]cholesterol efflux from HCAECs by 71.7% but did not mediate cholesterol efflux from HUVEC (Fig. 2*A*). These findings were similar to O'Connell *et al.* (21), and the reason for this observation may be the low ABCA1 expression levels in HUVECs.

Next, we treated ECs with increasing CCL2 doses (0–80 ng/ml) for 18 h or with increasing durations (0–24 h) at a fixed dose of 40 ng/ml CCL2. CCL2 decreased the [^3^H]cholesterol efflux to HDL in HCAECs and HUVECs from 15.3 and 12.1% at 20 ng/ml up to 52.6 and 39.1% at 80 ng/ml, respectively (Fig. 2*B*). Consistently, CCL2 decreased the [^3^H]cholesterol efflux to HDL in HCAECs and HUVECs by 28.4 and 25.1% at 12 h up to 68.7 and 48.6% at 24 h, respectively (Fig. 2*C*). Nevertheless, CCL2 decreased the [^3^H]cholesterol efflux to apoA1 by 15.9% up to 44.4% from 20 to 80 ng/ml and by 30.6% up to 55.7% from 12 to 24 h only in HCAECs. However, in HUVECs, the [^3^H]cholesterol efflux to apoA1 was barely detected and not induced by CCL2 (Fig. 2, *D* and *E*). Therefore, in HCAECs, CCL2 decreased the [^3^H]cholesterol efflux to both HDL and apoA1. In HUVECs, almost no [^3^H]cholesterol efflux to apoA1 and no effect of CCL2 could be observed. These data indicate that HCAECs are more sensitive than HUVECs regarding CCL2-induced suppression of cholesterol efflux. Additionally, endogenous CCL2 induced by TNF-α (10 ng/ml for 24 h) could significantly inhibit endothelial [^3^H]cholesterol efflux to HDL/apoA1 (data not shown).

##### CCL2 Suppressed HDL Internalization via CCR2 and Not CCR3 or CCR5

Many studies have indicated that CCR2, CCR3, and CCR5 are expressed in ECs and are the candidate receptors for CCL2. We next investigated the protein expression of these three receptors after CCL2 treatment, together with their impact on HDL uptake by HCAECs.

**FIGURE 3.**
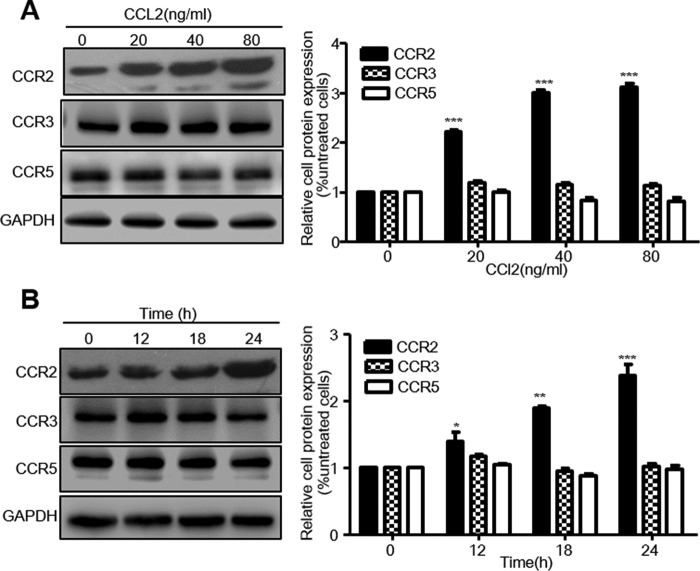
**CCL2 induced CCR2 expression in HCAECs.** Serum-starved HCAECs were treated with either increasing concentrations of CCL2 (0, 20, 40, and 80 ng/ml) in ECM containing 0.5% BSA for 18 h or with 40 ng/ml CCL2 for indicated times (0, 12, 18, and 24 h). Total proteins were extracted from the cultured cells, and the protein expressions of CCR2, CCR3, and CCR5 were detected using Western blotting as described under “Experimental Procedures.” The quantitative analysis of protein was performed using ImageJ software. *A,* CCR2, CCR3, and CCR5 protein expression of dose effect induced by CCL2. *B,* CCR2, CCR3, and CCR5 protein expression of time effect induced by CCL2. The experiment was performed three times. *, *p* < 0.05; **, *p* < 0.01; ***, *p* < 0.001 compared with the untreated cells.

After HCAECs were treated with an increasing concentration of CCL2 (0–80 ng/ml) at 18 h or with increasing durations (0–24 h) at a fixed dose of 40 μg/ml CCL2, the expression of CCR2, CCR3, and CCR5 proteins was detected by Western blotting. Only CCR2 expression was significantly increased in a dose- and time-dependent manner and by a maximum of 212 and 139%, respectively, after treatment with CCL2 at 80 μg/ml for 18 h and at 40 μg/ml for 12 h. In contrast, CCL2 did not increase or only slightly increased CCR3 or CCR5 expression (Fig. 3, *A* and *B*).

**FIGURE 4.**
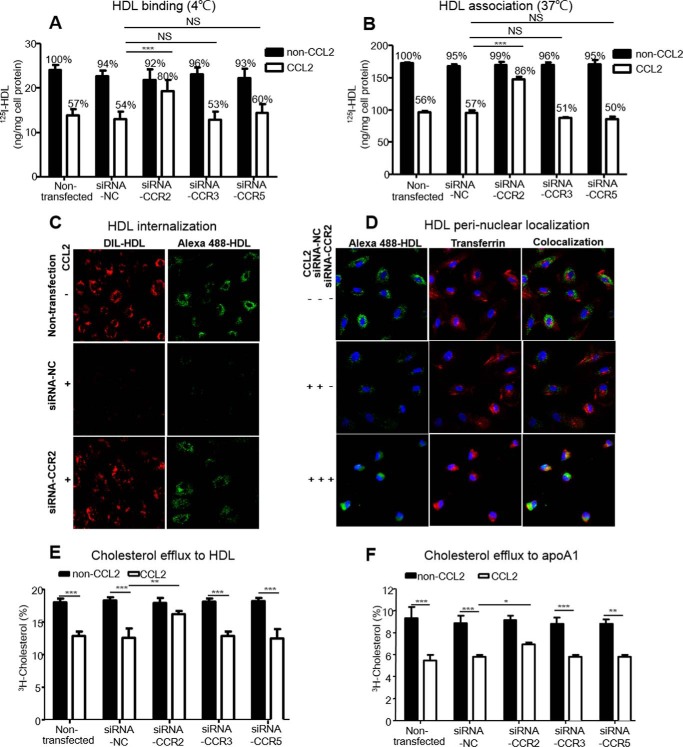
**siRNA-*CCR2* reversed CCL2 suppression on HDL binding, association, and internalization and on cholesterol efflux to HDL/apoA1 in HCAECs.** After using si-*CCR2*, si-*CCR3*, and si-*CCR5* interference, HCAECs were induced with 40 ng/ml CCL2 for 18 h. ^125^I-HDL binding at 4 °C, ^125^I-HDL association at 37 °C, HDL internalization, and [^3^H]cholesterol efflux were determined as the method in Figs. 1 and 2. *A,* roles of chemokine receptors on ^125^I-HDL binding after CCL2 was induced. *B,* roles of chemokine receptors on ^125^I-HDL association after CCL2 was induced. *NS,* non-significance. *C*, roles of chemokine receptors on the internalization of HDL phospholipid and protein after CCL2 was induced (×20). *D,* roles of chemokine receptors on the perinuclear localization of HDL protein after CCL2 was induced. The details of the method were same as used in Fig. 1*D* (×20). *E,* roles of chemokine receptors on [^3^H]cholesterol efflux to HDL after CCL2 was induced. *F,* roles of chemokine receptors on [^3^H]cholesterol efflux to apoA1. Non-transfected represents non-transfected cells, and *NC* represents negative control (NC)-transfected cells. The values were shown as the means ± S.D. of triplicate. *, *p* < 0.05; **, *p* < 0.01; ***, *p* < 0.001 compared with the indicated group.

The roles of CCR2, CCR3, and CCR5 in HDL uptake by HCAECs were further assessed by RNA interference (data not shown). After siRNA-*CCR2*, siRNA-*CCR3*, and siRNA-*CCR5* in HCAECs, we measured ^125^I-HDL binding at 4 °C and cell association at 37 °C with or without CCL2 (40 ng/ml, 18 h). In the presence of CCL2, siRNA-*CCR2* could increase ^125^I-HDL binding by 30% and cell association by 23% compared with those in siRNA-negative control (NC)-transfected cells; however, this reversed effect could not be observed by siRNA-*CCR3* and siRNA-**CCR5**. Therefore, CCL2 exerted its inhibitory effect on ^125^I-HDL binding and cell association in HCAECs through CCR2 but not CCR3 or CCR5 (Fig. 4, *A* and *B*).

Next, we found that the CCL2-induced suppression on the internalization of DIL-HDL phospholipid, and Alexa 488-HDL protein was reversed after siRNA-*CCR2* compared with those in siRNA-NC-transfected cells as detected by confocal microscopy (Fig. 4*C*). We further found that the internalized Alexa 488-HDL protein, by which perinuclear localization co-localized with Alexa 594-transferrin, increased after siRNA-*CCR2* in the presence of CCL2 (Fig. 4*D*). These data indicated that knocking down *CCR2* effectively reversed the CCL2-inhibited internalization of HDL protein and phospholipid.

##### CCL2 Regulated the [^3^H]Cholesterol Efflux to HDL/ApoA1 in HCAECs via CCR2

We further explored the roles of the three chemokine receptors on cholesterol efflux to HDL/apoA1 in HCAECs. After HCAECs were transfected with siRNA-*CCR2*, [^3^H]cholesterol was loaded for 24 h. Cholesterol efflux was initiated by 50 μg/ml HDL (Fig. 4*E*) or 20 μg/ml apoA1 (Fig. 4*F*) in the presence or absence of CCL2. In the presence of CCL2, siRNA-*CCR2* resulted in significant increases in the [^3^H]cholesterol efflux to HDL/apoA1 of 28.8 and 20.7%, respectively, compared with those in siRNA-NC-transfected cells. Similarly, *CCR2* knockdown in HUVECs significantly reversed the CCL2-induced inhibition of the cholesterol efflux to HDL by 27.8% (data not shown). These data indicate that CCR2 is essential for the suppression of HDL internalization and cholesterol efflux induced by CCL2 in HCAECs.

##### p42/44 MAPK Inhibition Reversed CCL2 Suppression on HDL Binding, Association and Internalization, and on [^3^H]Cholesterol Efflux in HCAECs

p42/44 MAPK signaling plays a pivotal role in various pathologies, including endothelial proliferation, antioxidant effects, and cardiovascular disease (22). Related studies showed that the inhibition of p42/44 MAPK significantly increased macrophage cholesterol efflux (23) and reduced hepatocyte DIL-HDL uptake (24). However, the role of the p42/44 MAPK pathway in CCL2-induced inhibition of HDL internalization and [^3^H]cholesterol efflux is unclear.

**FIGURE 5.**
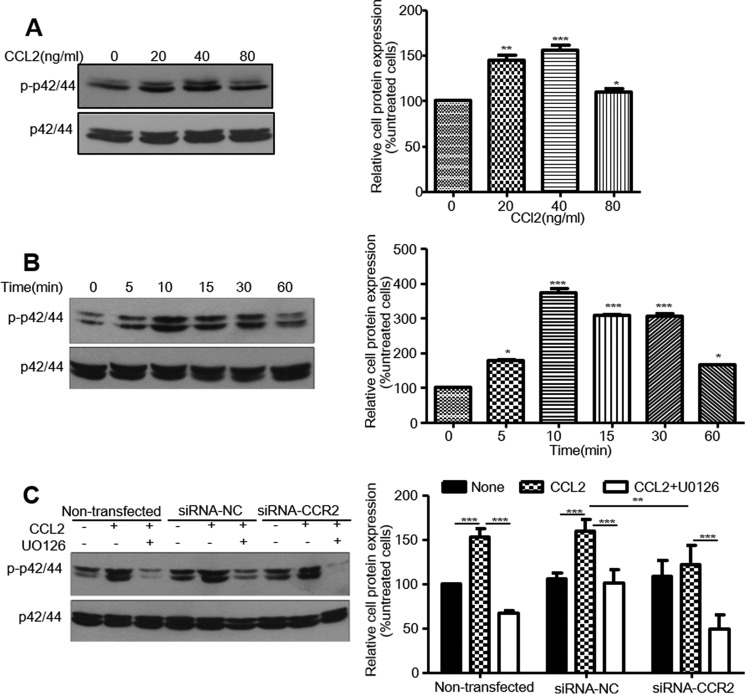
**CCL2 induced p42/44 MAPK (MAPK-ERK1/2) phosphorylation via CCR2 in HCAECs.** Starved HCAECs were treated with either increasing concentrations of CCL2 (0–80 ng/ml) for 10 min or with 40 ng/ml CCL2 for the indicated times (0–60 min). Proteins were extracted from the cultured cells, and the changes in phosphorylation of p42/44 MAPK were analyzed by Western blotting as described under “Experimental Procedures.” The quantitative analysis of protein was performed using ImageJ software. First, the ratios of phosphorylated MAPK/total MAPK at each time point were calculated. Second, the percentage of phosphorylation at 5, 10, 15, 30, and 60 min was calculated as the ratio of phosphorylated MAPK at each time point/the ratio of zero time point. *A,* p-p42/44 protein expression of dose-effect induced by CCL2. *B,* p-p42/44 protein expression of time effect induced by CCL2. *C,* p42/44 MAPK inhibitor could block the phosphorylation of p42/44 MAPK induced by CCL2 via CCR2. Cells transiently expressing control siRNAs (*siRNA-NC*) or *CCR2* siRNAs (*siRNA-CCR2*) were pretreated with 10 μm U0126 (a p42/44 MAPK inhibitor) for 30 min and then induced by 40 ng/ml CCL2 for 18 h. p-p42/44 protein expression were measured by Western blotting. The experiment was performed three times. *, *p* < 0.05; **, *p* < 0.01; ***, *p* < 0.001 compared with the untreated cells or the indicated groups.

First, we found that CCL2 treatment from 20 μg/ml up to the maximum of 40 μg/ml and from 5 min up to the maximum of 10 min resulted in an increase of p42/44 MAPK phosphorylation (Fig. 5, *A* and *B*). Additionally, this increase of p42/44 MAPK phosphorylation induced by CCL2 could be both blocked by pretreatment with siRNA-*CCR2* and U0126, a specific pharmacological inhibitor of p42/44 MAPK (Fig. 5*C*), suggesting that p42/44 MAPK could be activated by CCL2 via CCR2 induction.

**FIGURE 6.**
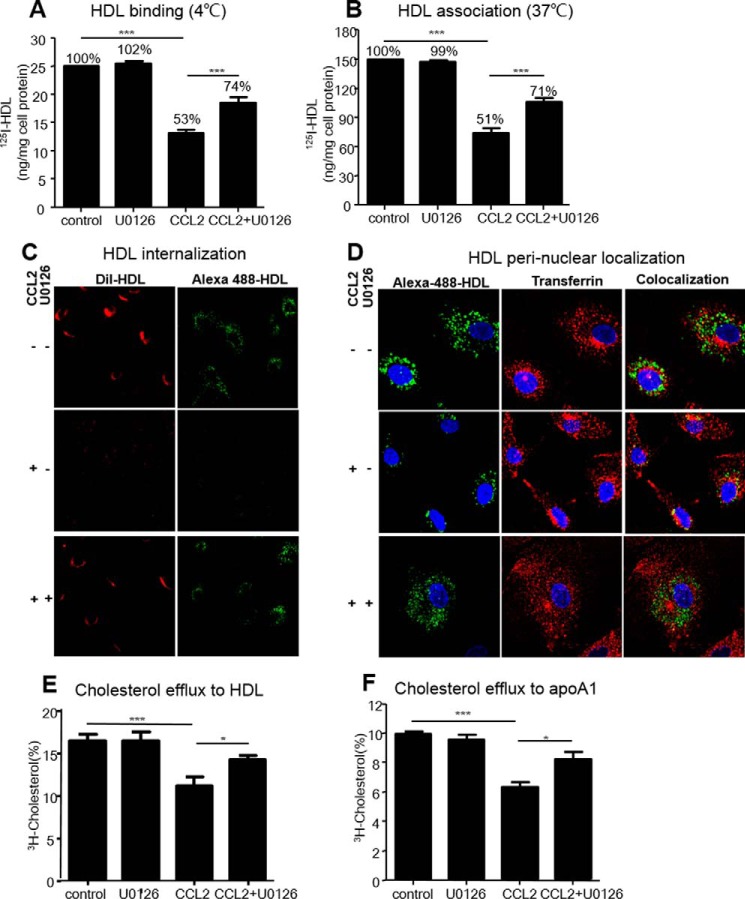
**p42/44 MAPK inhibition reversed CCL2 suppression on HDL binding, association, and internalization and [^3^H]cholesterol efflux to HDL/apoA1 in HCAECs.** HCAECs were preincubated with 10 μm U0126 for 30 min and then treated with 40 ng/ml CCL2 for 18 h. ^125^I-HDL binding at 4 °C, ^125^I-HDL association at 37 °C, HDL internalization, and [^3^H]cholesterol efflux were determined as the method in Figs. 1 and 2. *A,* effect of U0126 on the decrease of ^125^I-HDL binding at 4 °C induced by CCL2. *B,* effect of U0126 on the decrease of ^125^I-HDL association at 37 °C. *C,* effect of U0126 on the suppression of the internalization of HDL phospholipid and protein (×20). *D,* effect of U0126 on the suppression of perinuclear distribution of HDL protein (×63). *E,* effect of U0126 on the suppression of [^3^H]cholesterol efflux to HDL. *F,* effect of U0126 on the decrease of [^3^H]cholesterol efflux to apoA1. The results are represented as means ± S.D. of at least three individual experiments. *, *p* < 0.05; ***, *p* < 0.001 compared with the indicated group.

Next, we determined whether the suppression of CCL2 on the HDL internalization in ECs could be also reversed via p42/44 MAPK activation. In U0126-pretreated HCAECs, CCL2 only decreased ^125^I-HDL binding and cell association by 25.7 and 29.5%, respectively. However, in non-U0126-pretreated HCAECs, CCL2 decreased ^125^I-HDL binding and cell association by 47.0 and 50.4% compared with the control (Fig. 6, *A* and *B*). Similarly, pretreatment with U0126 also improved the suppression on the internalization of DIL-HDL phospholipid and Alexa 488-HDL protein (Fig. 6*C*). We also traced the reversed effect of internalized Alexa 488-HDL protein in the perinuclear area by U0126 pretreatment. The HDL protein localized in the perinuclear area, which was suppressed by CCL2, could be effectively reversed after U0126 pretreatment (Fig. 6*D*).

We further investigated the reverse effect of p42/44 MAPK inhibitor on [^3^H]cholesterol efflux to HDL/apoA1. After HCAECs were pretreated with U0126 for 30 min, CCL2 decreased the [^3^H]cholesterol efflux to HDL and apoA1 by 13.9 and 18.0%, respectively. Nevertheless, in non-U0126-pretreated HCAECs, CCL2 decreased the [^3^H]cholesterol efflux to HDL and apoA1 by up to 32.1 and 36.7%, respectively, compared with the control (Fig. 6, *E* and *F*). Analogous results were obtained in HUVECs (data not shown). These data indicated an important role for p42/44 MAPK signaling in CCL2-induced HDL internalization and cholesterol efflux in ECs.

## Discussion

CCL2 is regarded as a risk factor for the initiation and progression of atherosclerosis and for patients with CHD. Exploring the impairing mechanism of RCT in the initial stage will provide novel strategies of anti-atherosclerosis at the early step. Our previous study suggested that CCL2 decreased RCT in hepatocytes (20). The major focus of this study was investigating the effect of CCL2 on HDL function involving the initial regulation of RCT in ECs.

A large number of studies have reported that high CCL2 levels in patients with CHD are associated with an increased risk of long-term mortality and major adverse cardiac outcomes (11, 25). Additionally, a previous study by Karabacak *et al.* (26) reported a similar correlation in isolated patients with low HDL-C levels. However, recent clinical trials have failed to demonstrate a significant clinical benefit despite increases in HDL levels (27, 28). In our study, plasma HDL2 levels were decreased, whereas CCL2 levels were increased, and CCL2 was negatively correlated with HDL2. We hypothesized that the maturation process of HDL was attenuated by CCL2. Indeed, an increasing body of evidence has indicated that both acute and chronic inflammatory conditions impair the lipid homoeostasis-regulating and anti-inflammatory properties of HDL and can turn HDL into a proinflammatory molecule (29). Tehrani *et al.* (30) reported that the protective effect of HDL-C on CHD is attenuated when levels of inflammation are high, even with elevated HDL-C. Thus, we considered that the biological function of HDL and the inflammatory status of the population might be of great importance for determining the clinical benefits of HDL-elevating therapies.

**FIGURE 7.**
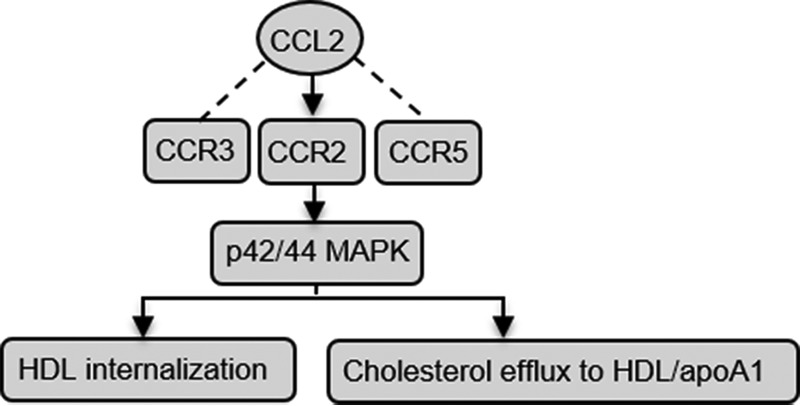
**Proposed model for the impact of CCL2 on HDL internalization and cholesterol efflux and regulatory mechanisms in HCAECs.** CCL2 binds to CCR2, activates p42/44 MAPK pathway, and leads to the suppression of HDL internalization and cholesterol efflux to HDL/apoA1. *ApoA1*, apolipoprotein AI.

Intriguingly, we found that CCL2 suppressed ^125^I-HDL binding, cell association, HDL phospholipid and protein internalization, and [^3^H]cholesterol efflux in HUVECs and HCAECs. Our data showed CCR2 was activated significantly, whereas CCR3/5 was not induced by CCL2 and participated in the above process. This suggested CCL2 exhibited a particular affinity for CCR2. Further study suggested that the p42/44 MAPK pathway participated in the CCL2-induced attenuation of HDL function via CCR2 in ECs (Fig. 7).

The endothelium lines the vasculature as a single layer of ECs. Because ECs represent a semipermeable barrier, changes in EC functions appear to play a key role in the pathogenesis of atherosclerosis. Generally, transendothelial translocation of protein occurs by paracellular and transcellular pathways. A previous study (6) and our study demonstrated that ECs bound and associated with ^125^I-HDL in a concentration- and temperature-dependent manner. Simultaneously, this present study demonstrated that ECs internalized HDL, which localized in perinuclear area similar to the findings of Fruhwürth *et al.* (31) Notably, not all cells internalize HDL, although they express HDL receptors (32). Importantly, the majority of the HDL internalized by ECs is not degraded but re-secreted as intact proteins (6). Inflammatory cytokines modulate the transendothelial transport of proteins. For instance, Wang *et al.* (34) suggested a reduction in insulin transport after IL-6 stimulation, whereas Robert *et al.* (35) demonstrated that IL-6 enhanced the capacity of ECs to bind, associate, and transport HDL. However, to the best of our knowledge, we observed that CCL2 inhibited the binding and association of ^125^I-HDL with ECs. Additionally, CCL2 qualitatively suppressed HDL phospholipid and protein internalization. Cholesterol efflux is the key step in RCT, and acute inflammation impairs the cholesterol efflux capacity (36, 37). However, Frisdal *et al.* (38) demonstrated that IL-6 promoted cholesterol efflux in human macrophages and attenuated the proinflammatory response. Thus, although IL-6 is primarily considered a major proinflammatory cytokine, it may also act as a key anti-inflammatory mediator to control inflammatory responses (39). Interestingly, our findings showed that exposure to a low dose of CCL2 for a short duration efficiently decreased the endothelial cholesterol efflux to HDL and apoA1. This result is consistent with our previous study, which suggested that CCL2 suppressed HDL lipid uptake and cholesterol efflux in HepG2 cells (20). This study illustrates that CCL2 is a key mediator in HDL metabolism. However, an obvious difference in cholesterol efflux was found between HCAECs and HUVECs. In HCAECs, apoA1 and HDL efficiently mediate cholesterol efflux, whereas only HDL mediates the cholesterol efflux in HUVECs. ABCA1 expression most likely differs between HCAECs and HUVECs. Fig. 2*B* illustrates that HCAECs are more receptive to CCL2 than HUVECs. Thus, HCAECs may correspond better with CAD models and are more likely to be damaged by inflammatory factors.

Our data also suggested that unlike exogenous CCL2, endogenous CCL2 induced by TNF-α could only decrease cholesterol efflux to HDL/apoA1 but had no effect on HDL binding and association in HCAECs. The possible reason for this result was that TNF-α could induce multiple cytokines and chemokines (CCL2, CCL5, ICAM-1 VCAM-1, CXCL10, and IL6) (40, 41), which have a competing effect on HDL metabolism, like IL-6. It is reported that IL-6 increases the endothelial binding and association of HDL through induction of endothelial lipase (35). Moreover, the effect of other cytokines and chemokines on HDL metabolism is not fully clear.

Sviridov *et al.* (42) reported an antibody against the HDL-binding protein enhanced HDL uptake that was not accompanied by parallel changes in cholesterol efflux in rat hepatoma cells. In contrast, Pagler *et al.* (43) demonstrated the involvement of HDL uptake and re-secretion in the maintenance of cholesterol homeostasis in cases of disturbed intracellular cholesterol efflux. The authors also showed that SR-BI mediated HDL endocytosis, leading to cholesterol efflux (44). Moreover, Barlic *et al.* (45) found that CXCL16 promoted HDL uptake and cholesterol release in human macrophages. These studies suggested that HDL uptake was accompanied by cholesterol efflux. In our study, CCL2 decreased HDL internalization, leading to a reduction in cholesterol efflux from HCAECs. Based on our results, HDL internalization in ECs was closely related to cholesterol efflux, and CCL2 mediated the parallel inhibition of HDL internalization and cholesterol efflux. HDL internalization in the endothelium had two principle physiological roles. First, the interaction of HDL with ECs mediates cholesterol efflux. Second, HDL crossing the ECs is the first step that enables access to foam cells (6). The latter role represents the anti-atherosclerotic effect of HDL. Vascular ECs are the most resistant to cholesterol accumulation of the cells that compose the atherosclerotic plaque. Efficient cholesterol efflux from HCAECs also has the following two protective effects: maintaining cholesterol homeostasis and endothelial function (21) and playing a vascular protective role by minimizing the amount of cholesterol entering the vessel intima (7). Therefore, we propose that CCL2 is a risk factor for atherosclerosis through the impairment of endothelial HDL uptake and cholesterol efflux.

Typically, chemokines exert their roles through specific receptors. CCL2 has three candidate receptors: CCR2, CCR3, and CCR5. CCR5 is expressed by atheroma-associated cells, including vascular ECs, and favors the development of an inflammatory response and atherosclerosis in LDL-knock-out mice (18). Few studies have analyzed the role of CCR3 in atherosclerosis. We found that CCR2, but not CCR3 or CCR5, participated in HDL metabolism, thus representing a potential chemokine axis of atherosclerosis. A number of studies have demonstrated that genetic deletion of CCL2 or CCR2 slowed the disease course in animal models of atherosclerosis (46, 47). CCL2-CCR2 signaling is thought to play an exacerbating role in atherosclerosis presumably via the recruitment of inflammatory monocytes to the site of atherosclerotic plaques (48). However, we analyzed the role of CCL2-CCR2 in the development of atherosclerosis through a new view of HDL metabolism. The roles of chemokines and their receptors in controlling several physiological and pathological processes have only become evident in the last couple of years. Views on chemokine receptor activation have switched to the regulation of pleiotropic signaling pathways that influence numerous molecular and cellular processes (49). Therefore, we explored the role of the p42/44 MAPK pathway on HDL uptake and cholesterol efflux induced by CCL2. The extensively studied MAPK family, including p42/44, p38, JNK, and ERK5, demonstrates unique intracellular signaling mechanisms that influence the signaling of cardiac development, metabolism, and pathogenesis (50). Through p42/44 MAPK, CCL2 enhances the survival and invasiveness of endometrial stromal cells and coordinates the survival and motility of breast cancer cells (51). Another study showed that p42/44 MAPK inhibition enhanced the PPARα-dependent degradation of SR-BI in hepatocytes, leading to a reduction in DIL-HDL uptake (24). Other studies also demonstrated that ERK1/2 inhibition by U0126 induced macrophage cholesterol efflux, activated RCT, and reduced atherosclerotic lesions in apoE-deficient mice (23, 52). Our findings showed that CCL2 induced p42/44 MAPK phosphorylation via CCR2. Moreover, the inhibition of p42/44 MAPK improved the suppression of HDL uptake and cholesterol efflux induced by CCL2. Our findings provide a potential mechanism by which CCL2 opposes the atheroprotective role of HDL.

The endothelium is the orchestral conductor of blood vessel function affected by endothelial cell proliferation, migration, and apoptosis (53). Notably, the reduction in proliferation and increase in apoptosis in ECs induced by CCL2 impact cholesterol metabolism (54, 55). Therefore, we hypothesize that CCL2 suppresses HDL uptake and cholesterol efflux by regulating endothelial proliferation and apoptosis, which we will study next. Cellular cholesterol efflux and RCT constitute a potent physiological protective system against atherosclerosis (33, 56). However, the involvement of many factors in HDL metabolism makes this system a difficult therapeutic target. Our study establishes a link between CCL2, HDL, and endothelial cholesterol efflux and provides the first experimental evidence for the p42/44 MAPK-dependent mechanism of atherosclerosis mediated by CCL2 at the molecular and cellular levels using primary ECs relevant to atherosclerotic plaques. Our study has proposed important new questions for future investigations. For instance, pharmacologically blocking CCL2-CCR2 signaling appears to be a viable strategy for treating atherosclerosis and is currently being pursued in clinical trials.

## Experimental Procedures

### 

#### 

##### Recruitment of Patients with CAD and Healthy Subjects and Collection of Blood Samples

Two hundred and thirty six patients with angiographically diagnosed CAD (at least 50% obstruction of one or more major coronary arteries) and 241 healthy controls were recruited from Sun Yat-sen Memorial Hospital of Sun Yat-sen University (Guangzhou, China). A medical history and record revealed CAD risk factors, including diabetes, hypertension, hypercholesterolemia history, and cigarette smoking. Patients with hypothyroidism, renal dysfunction, or hepatic failure were excluded from the study. None of the patients participated in lipid-lowering therapy before blood collection. Blood was drawn after an overnight fast and collected in EDTA-coated tubes. The protocol was approved by the institutional ethics committee of the hospital.

An Olympus AU-640 (Germany) was used for lipid analysis, and ELISA (BMS281TEN, Austria) was used for measuring CCL2. The levels of HDL particles were measured as follows. First, HDL-C levels were measured in serum before separation. The serum samples (300 μl) were precipitated with heparin containing MnCl_2_ and dextran sulfate and separated by centrifugation at 10,000 rpm for 10 min. The amounts of HDL3 in the supernatant were measured using homogeneous HDL-EX HDL-C assays (Denka Seiken, Tokyo, Japan). Levels of HDL2 were derived from the following formula: HDL2 = HDL-C − HDL3.

##### Cell Culture and Treatment

HCAECs (ScienCell; catalogue number 6020) and HUVECs (ScienCell; catalogue number 8000) were cultured in flasks coated with fibronectin (2 μg/cm^2^) in endothelial cell medium (ECM, ScienCell; catalogue number 1001) containing 1% endothelial cell growth supplement, 5% fetal bovine serum, and 1% penicillin/streptomycin solution in a humidified atmosphere containing 5% CO_2_ and 95% air at 37 °C. Passages 4–8 were used for the experiments. The cells were grown until 80–90% confluent and then incubated in serum-free medium containing 0.5% bovine serum albumin (BSA) for 6 h. To investigate the dose effect of CCL2, the cells were incubated in serum-free medium containing 0.5% BSA with CCL2 (R&D Systems; catalogue number 279-MC-010) at different concentrations (0–80 ng/ml) for 18 h. Cells treated with 40 ng/ml CCL2 for increasing times (0–24 h) were used to study the time-dependent effects of CCL2. To examine the involvement of p42/44 MAPK, we first pretreated the cells with the MAPK inhibitor U0126 (10 μm; catalogue number 9903; Cell Signaling Technology) for 30 min. The cells were then incubated with or without CCL2 (40 ng/ml) for 18 h.

##### Isolation and Labeling of High Density Lipoproteins

Plasma was collected from healthy volunteers, and HDL was prepared by sequential ultracentrifugation (*d* = 1.21 g/ml). HDL was iodinated in the presence of ^125^I (Amersham Biosciences) by the iodine monochloride method. HDL phospholipid was labeled with DIL (Biomedical Technologies Inc., Stoughton, MA), and protein was labeled with Alexa 488, according to the manufacturer's instructions. The HDL was stored at 4 °C under N_2_ gas.

##### Binding (4°C) and Association (37 °C) of ^125^I-HDL to/with ECs

Binding and association of ^125^I-HDL assays were performed as described elsewhere (6). Briefly, ECs were seeded into 12-well dishes at a concentration of 3 × 10^5^ cells/well for HDL binding studies after CCL2 treatment. The assays were performed in ECM containing 10 μg/ml ^125^I-HDL (or the indicated amount) in the absence (total binding/cell association) or in the presence (nonspecific binding/cell association) of a 40-fold excess of unlabeled HDL. After incubation at 4 °C for 1 h, the cells were solubilized in 0.1 n NaOH for 60 min at room temperature, and the protein concentration and radioactivity were measured in the lysate. Radioactivity was measured by using a counter from PerkinElmer Life Sciences, and the measurements were normalized to the protein content determined by the Bradford protein assay (Bio-Rad). Specific binding was determined by subtracting the values obtained in the presence of excess unlabeled HDL (nonspecific) from the binding obtained in the absence of the unlabeled HDL (total). The results were shown as specific binding. The protocol for the 37 °C cell association studies was identical to the 4 °C protocol except that the cells were incubated with ^125^I-HDL at 37 °C for 1 h.

##### Internalization of HDL Protein and Phospholipid

To investigate HDL phospholipid and protein internalization, HCAECs were cultured in glass-bottom cell culture dishes until 80–90% confluence. Then cells were incubated with DIL-HDL and Alexa 488-HDL for 1 h at 37 °C, respectively. Next, the cells were washed six times for 5 min in cold phosphate-buffered saline with Tween 20 (PBST) and fixed in 3% paraformaldehyde for 30 min. Finally, the samples were examined using a confocal microscope (LSM780). To investigate the perinuclear cellular distribution of HDL, the cells were incubated for 30 min with 10 μg/ml HDL labeled with Alexa 488 and 50 μg/ml Alexa 594-transferrin (Life Technologies, Inc.; catalogue number T-13343). After washing and fixation, the cells were incubated with DAPI for 5 min. Then, the cells were washed three times for 10 min and examined using a confocal microscope

##### Cholesterol Efflux to HDL+/ApoA1 Assay

After 6 h of serum starvation, the cells were washed with PBS and labeled by incubation in ECM supplemented with 0.5% BSA containing [^3^H]cholesterol (1 μCi/ml; PerkinElmer Life Sciences) for 24 h. Cellular cholesterol efflux was initiated by the addition of ECM containing 0.2% BSA with 20 μg/ml human apoA1 or 50 μg/ml HDL in the presence or absence of CCL2 (40 ng/ml). After incubation, the radioactivity of the medium and cells was measured with a liquid scintillation counter. The cholesterol efflux was expressed as the percentage of counts in the medium relative to the total counts for the medium and cells together. The final cholesterol efflux was calculated as the percentage of total [^3^H]cholesterol released into the medium after subtraction of the values obtained in the absence of apoA1 or HDL.

##### Western Blotting Analysis

Following incubation, the cells were harvested, washed with PBS (pH 7.4), and lysed in RIPA buffer (Roche Applied Science) for 30 min at 4 °C. The proteins were fractionated on 4–10% gradient SDS-polyacrylamide gels and electrophoretically transferred to polyvinylidene difluoride (PVDF) membranes (Invitrogen). The membranes were incubated with a Tris-buffered saline (TBS) blocking solution (200 mm Tris-HCl, 150 mm NaCl, and 5% nonfat dry milk) for 1 h at room temperature. The membranes were immunoblotted overnight at 4 °C with the appropriate antibody as follows: rabbit polyclonal anti-CCR2 antibody (Abcam; diluted 1:1,000); rabbit monoclonal anti-CCR3 antibody (Abcam; diluted 1:500); mouse monoclonal anti-CCR5 antibody (Abcam; diluted 1:1,000); anti-p42/44 MAPK antibody (Cell Signaling Technology; diluted 1:1,000); phospho-p42/44 MAPK antibody (Cell Signaling Technology; diluted 1:2,000); or rabbit monoclonal anti-GAPDH antibody (Abcam; diluted 1:10,000). After three washes with TBS containing 0.1% Tween 20, the membranes were incubated with horseradish peroxidase-conjugated secondary antibody for 1 h at room temperature. To visualize the immunoreactive bands, enhanced chemiluminescence (ECL) Western blotting detection reagents and medical x-ray films were used according to the manufacturers' instructions. The band intensity was analyzed with ImageJ software, and the expression data were normalized to GAPDH expression.

##### siRNA Transfection

*CCR2, CCR3,* or *CCR5* were knocked down in HCAECs using short interfering RNA (siRNA) oligonucleotides (Gene Pharma, Shanghai, China) targeted to the cDNA sequences of the human *CCR2, CCR3*, and *CCR5* genes, respectively. The siRNA sequences were as follows: *CCR2*, 5′-GCTGCAAATGAGTGGGTCTTT-3′; *CCR3*, 5′-CCCAGAGGATACAGTATAT-3′; *CCR5*, 5′-GTGTCAAGTCCAATCTATG-3′; and si-control, 5′-UUCUCCGAACGUGUCACGUTT-3′. A total of 1.0 × 10^6^ cells were transfected with 50 pmol of siRNA/well in a 6-well plate or 5 × 10^5^ cells with 12.5 pmol of siRNA/well in a 24-well plate by using Lipofectamine RNAiMax (Invitrogen) according to the manufacturer's instructions. Protein was collected, and cholesterol efflux and HDL uptake assays were performed 48 h after the siRNA transfection.

##### Statistical Analysis

All of the data are expressed as the means ± S.D. To compare continuous variables, the statistical significance of differences was determined using Student's *t* test, non-parametric test, or one-way analysis of variance followed by Bonferroni's post hoc test, as appropriate. Categorical variables were compared using the χ^2^ test. Simple correlation analysis and multiple regression analysis of the association between CCL2 and HDL2 after adjustment of potential confounders were performed by SPSS 14.0 software. Statistical significance was defined as a two-tailed probability of less than 0.05.

## Author Contributions

R.-L. S. conducted most of the experiments, analyzed the results, and wrote most of the paper. J.-L. B. helped R.-L. S. conduct experiments labeling HDL with ^125^I. C.-X. H. helped R.-L. S. conduct experiments of cholesterol efflux. J.-Y. J. and B. Z. helped collect blood samples of CAD patients and healthy controls. S.-X. Z. helped conduct experiments searching for regulatory mechanisms of cholesterol metabolism. W.-B. C. helped to replenish the experiments. H.-W. helped to revise the manuscript and improve the tables and figures. Y.-L. Z. and J.-F. W. conceived the idea for the project and wrote the paper with R.-L. S.

## Footnotes

3 The abbreviations used are:

HDL-C: HDL cholesterol
CCL2: CC-chemokine ligand 2
CCR2: CC-chemokine receptor 2
CCR3: CC-chemokine receptor 3
CCR5: CC-chemokine receptor 5
HCAEC: human coronary artery endothelial cell
HUVEC: human umbilical vein endothelial cell
RCT: reverse cholesterol transport
CAD: coronary artery disease
CHD: coronary heart disease
SR-BI: scavenger receptor class B, type I
DIL: 1,1′-dioctadecyl-3,3,3′,3′-tetramethylindocarbocyanine perchlorate
NC: negative control
ECM: endothelial cell medium
TC: total cholesterol
TG: triglyceride
BMI: body mass index.
